# The production method affects the efficacy of platelet derivatives to expand mesenchymal stromal cells in vitro

**DOI:** 10.1186/s12967-017-1185-9

**Published:** 2017-05-01

**Authors:** Martina Bernardi, Francesco Agostini, Katia Chieregato, Eliana Amati, Cristina Durante, Mario Rassu, Marco Ruggeri, Sabrina Sella, Elisabetta Lombardi, Mario Mazzucato, Giuseppe Astori

**Affiliations:** 1 Advanced Cellular Therapy Laboratory, Hematology Unit, Vicenza Hospital, Vicenza, Italy; 2 Hematology Project Foundation, Contrà S. Francesco 41, Vicenza, Italy; 3 Stem Cell Collection and Processing Unit, CRO National Cancer Institute-IRCCS Aviano, Aviano, Italy; 4 Department of Microbiology, San Bortolo Hospital, Via Rodolfi 37, 36100 Vicenza, Italy

**Keywords:** Platelet lysate, Platelet releasate, Mesenchymal stromal cells, Mesenchymal stem cells, Ex vivo expansion, Fetal bovine serum

## Abstract

**Background:**

The use of fetal bovine serum (FBS) as a media supplement for the ex vivo expansion of bone-marrow derived mesenchymal stromal cells (BM-MSC) has been discouraged by regulatory agencies, due to the risk of transmitting zoonoses and to elicit immune reactions in the host once transplanted. Platelet derivatives are valid FBS substitutes due to their content of growth factors that can be released disrupting the platelets by physical methods or physiological stimuli. We compared platelet derivatives produced by freezing/thawing (platelet lysates, PL) or after CaCl_2_ activation (platelet releasate surnatant rich in growth factors, PR-SRGF) for their content in growth factors and their ability to support the ex vivo expansion of BM-MSC.

**Methods:**

The cytokine content in the two platelet derivatives was evaluated. BM-MSC were expanded in complete medium containing 10, 7.5 and 5% PL or PR-SRGF and the cell phenotype, clonogenic capacity, immunomodulation properties and tri-lineage differentiation potential of the expanded cells in both media were investigated.

**Results:**

The concentration of PDGF-AB, PDGF-AA, PDGF-BB in PR-SRGF resulted to be respectively 5.7×, 1.7× and 2.3× higher compared to PL. PR-SRGF promoted a higher BM-MSC proliferation rate compared to PL not altering BM-MSC phenotype. Colony forming efficiency of BM-MSC expanded in PR-SRGF showed a frequency of colonies significantly higher than cells expanded in PL. BM-MSC expanded in PL or PR-SRGF maintained their immunomodulatory properties against activated lymphocytes even if BM-MSC expanded in FBS performed significantly better.

**Conclusions:**

The method used to release platelet factors significantly affects the enrichment in growth factors and overall product performance. The standardization of the production process of platelet derivatives and the definition of their release criteria requires further investigation.

## Background

Fetal bovine serum (FBS), rich in growth factors able to stimulate the cell proliferation is historically used for culturing cells [1, 2]. In 2008, the Committee for Medicinal Products for Human use of the European Medicine Agency discouraged the use of animal derivatives since they may harbor infectious agents and may possibly increase undesirable immunological responses in the recipient [3]. As a consequence, there is an urgent need to find a replacement for FBS for the production of safe and reproducible cell therapy products.

The use of platelet lysate (PL) for the ex vivo expansion of mesenchymal stromal cells (MSC) was originally proposed by Doucet et al. [4]. There are strong evidences that PLs are able to support the expansion of bone marrow (BM) [5, 6], umbilical cord blood [7, 8] and adipose tissue derived-MSC [9].

Release of growth factors from platelets can be obtained through physical methods by freezing-thawing the platelet rich plasma obtained from the platelet apheresis or from the buffy coat. Briefly, the platelet rich plasma bags are frozen overnight at −80 °C and then thawed at +37 °C; the cycle is repeated for 1–3 times. After pooling and one or more centrifugation/filtration steps in order to remove cellular debris, PL is ready for being added to the growth media [10].

Alternatively, platelets can be disrupted by sonication as formerly described by Hara et al. [11] and by our group [12].

Platelet factors can be also released by physiological stimuli as thrombin, collagen, adenosine diphosphate, epinephrine and thrombin receptor-activating peptide, CaCl_2_ or tri-n-buthyl phosphate in order to obtain the so-called platelet releasate (PR) [13, 14]. The method used to release the platelet factors could represent a variable able to influence the quality of the final product but only few studies have addressed this issue so far.

In this work we have produced PL by freezing/thawing and platelet releasate by CaCl_2_ activation in order to obtain the so-called platelet releasate surnatant rich in growth factors PR-SRGF [15]. The two platelet derivatives were compared for their content in selected growth factors. Moreover, BM-MSC were expanded in complete medium containing 10, 7.5 and 5% PL or PR-SRGF and the cell phenotype, clonogenic capacity, immunomodulation properties and tri-lineage differentiation potential of the expanded cells in both media were investigated.

## Methods

### Preparation of PL

Platelet apheresis were collected from 15 donors by using a Trima Accel separator (Caridian BCT Inc, Lakewood, CO, USA), transferred to 50-ml tubes (Falcon, Corning MA, USA) and stored at −80 °C. After 2 cycles of freezing/thawing the aliquots were centrifuged at 1600×*g* for 15 min at room temperature and the supernatants were collected, pooled, filtered using a 70 μm cell strainer (Falcon, Corning MA, USA) and finally stored at −20 °C until use.

### Preparation of PR-SRGF

Platelet apheresis were collected from 15 donors by using a multicomponent collection system (Haemonetics, MA, USA). Platelet activation was performed by adding CaCl_2_ (Monico, Venice, Italy) at the final concentration of 0.04 M and after incubation at 40 °C for approximately 60 min until complete clot formation. Bags were centrifuged for 5 min at 2200×*g* and the SRGF collected and stored at −80 °C.

### Cytokine determination

Platelet-derived growth factor (PDGF)-AA, PDGF-AB, PDGF-BB, epidermal growth factor (EGF), vascular endothelial growth factor (VEGF), basic fibroblast growth factor (FGF-basic), insulin like growth factor-1 (IGF-1) and transforming growth factor-β1 (TGF-β1) were quantified by using a Quantikine ELISA Kits (R&D Systems, Minneapolis, MN, USA).

### BM-MSC isolation and expansion

Total nucleated cells were isolated from the washout and filters used for bone marrow collection (n = 3). Cells were counted and plated at 100,000 cells/cm^2^ in low glucose Dulbecco’s modified Eagle’s medium + glutamax (Gibco Invitrogen, Carlsbad, CA, USA) containing penicillin/streptomycin (Sigma-Aldrich, Saint Louis, Missouri, USA) and heparin 30 U/ml (Hospira Italia, Napoli, I) supplemented with 10% FBS (Gibco Invitrogen, Carlsbad, CA, USA) or 7.5% platelet derivatives. Cells were incubated at 37 °C and 5% CO_2_ concentration. After 3–4 days in culture, non-adherent cells were removed and fresh medium was added (P0). The resulting plastic adherent cells were expanded until 80–90% confluence and then harvested with TrypLE™ Select (Gibco Invitrogen, Carlsbad, CA, USA) and counted (P0).

BM-MSC were thawed and expanded in presence of 10% FBS or 5, 7.5 and 10% PL or PR-SRGF. At each sub-cultivation, the population doubling (PD) was calculated as follows: PD = log10(N)/log10(2); where N is the number of cells harvested-the number of cells initially seeded. The cumulative PD (cPD) was calculated adding to the PD of the passage under analysis the PDs of the previous passages.

### Immunophenotypic analysis

Cells were stained at P4 and P7 following the International Society for Cellular Therapy guidelines [16] with anti-human antibodies against CD31, CD34, CD45, CD105, CD44, CD90 (all from Beckman Coulter, Fullerton, CA, USA) and CD73, (Beckton Dickinson, Franklin Lakes, NJ, USA). Briefly, about 1 × 10^5^ cells were incubated for 15 min at room temperature with the specific monoclonal antibody. At least 10,000 events were acquired by using a CYTOMICS FC500 flow cytometer (Beckman Coulter Fullerton, CA, USA).

### Immunomodulation

Immunomodulation analysis was performed by co-culturing BM-MSC with peripheral blood mononucleated cells (PBMC) isolated by density gradient centrifugation and labeled with Carboxyfluorescein Succinimidyl ester (Celltrace CFSE Cell Proliferation Kit, Invitrogen, Carlsbad, CA, USA) at the PBMC:BM-MSC ratio of 20:1, 10:1 and 5:1. PBMC were stimulated with 500 U/ml of Interleukin 2 (Proleukin, Novartis Pharma, Varese, I) and 0.5 µg/ml antibody anti CD3 (Miltenyi Biotec, Bergisch Gladbach, DE). PBMC unstimulated and activated in absence of effector cells were used as controls. After 6 days, PBMC were labeled with CD45 PC7 (Beckman Coulter Fullerton, CA, USA) and 7-aminoactinomycin D (7-AAD) (Invitrogen Carlsbad, CA, USA) and analyzed with a FC500 flow cytometer (Beckman Coulter Fullerton, CA, USA). The inhibition of the PBMC CD45+ 7AAD-cell subset induced by BM-MSC was expressed according to the following formula:

$$\frac{{(\% {\text{ CFSE}}^ + \; {\text{activated PBMC in absence of BM-MSC}}\, - \,\% {\text{CFSE}}^ + \; {\text{activated PBMC in presence of BM-MSC}}) \times 100}}{{\% \;{\text{CFSE}} ^+ \; {\text{activated PBMC in absence of BM-MSC}}}}$$

### BM-MSC tri-lineage differentiation

For osteogenic and adipogenic differentiation, BM-MSC at the end of passage 4 were seeded at a density of 4000 cells/cm^2^ on cell culture coverslips (Thermo Fisher Scientific, Waltham, MA, USA) arranged in 24-well plates (Falcon, Corning, NY, USA) in presence of medium supplemented with 10% FBS, 7.5% PL or 7.5% PR-SRGF. At 70–80% of cell confluence, the medium was replaced with specific differentiation media, then renewed every 3–4 days for 21 days. To induce adipogenic differentiation, cells were incubated using the StemPro adipogenic differentiation kit (Thermo Fisher Scientific, Waltham, MA, USA), according to the manufacturer’s instructions. The presence of intracellular lipid droplets was detected by standard staining with Oil Red O (Diapath, Bergamo, Italy), according to the manufacturer’s instructions. In parallel, cells were also grown using the StemPro^®^ osteogenic differentiation kit (Thermo Fisher Scientific, Waltham, MA, USA) to induce osteogenic differentiation. The presence of calcium deposit was evaluated by von Kossa staining (Sigma-Aldrich, Saint Louis, MO, USA). Cells were fixed with 10% formalin for 5 min at room temperature, incubated with 1% silver nitrate solution for 15 min and exposed to ultraviolet light for 2 h. Coverslips were rinsed with distilled water and 5% sodium thiosulfate to remove unreacted silver. Finally, cells were counterstained with Nuclear Fast Red Solution (Sigma-Aldrich, Saint Louis, MO, USA). To induce chondrogenesis, 10 × 10^4^ cells in 100 μl chondrogenic induction medium (StemPro chondrogenic differentiation kit, Thermo Fisher Scientific, Waltham, MA, USA) were placed in a 24-well plates (Falcon, Corning, NY, USA) and after an incubation at 37 °C for about 12 h, 700 μl chondrogenic induction medium were added. Fresh chondrogenic medium was added every 3–4 days. After 28 days, the micromass was fixed with formalin 10% and stained with Alcian Blue 1% acetic acid (Sigma-Aldrich, Saint Louis, MO, USA).

### Colony forming efficiency

At passage 2 (P2) and 5 (P5), 200 BM-MSC were plated in duplicate in 100-mm diameter culture dishes (Cellstar, Grainer Bio-One GmbH, Germany) in medium containing 10% FBS, 7.5% PL and 7.5% PR-SRGF respectively. The cell medium were changed weekly and after 14 days the cells were fixed with 10% formalin and stained with May-Grunwald Giemsa. Stained plates were scored for colony forming units (CFU) by an inverted light microscope (Axiovert 40 CFL, Zeiss, Germany). Colonies consisting of at least 30 cells were counted as CFU.

### Statistical analysis

The analysis of variance was calculated by using the two way ANOVA test by using the GraphPad Prism software (GraphPad Prism Software version 5.01, Inc. La Jolla, CA, USA).

## Results

### Cell proliferation

**Fig. 1:**
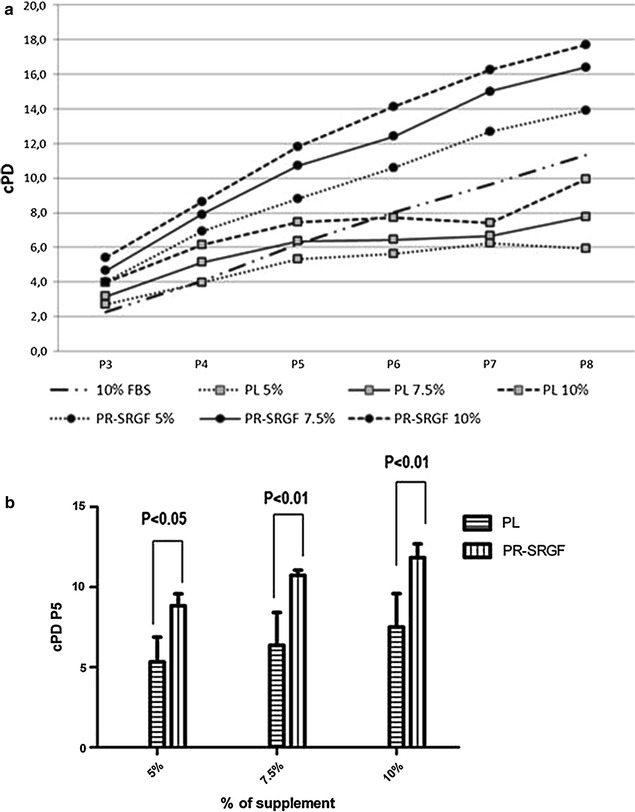
cPD of BM-MSC expanded in presence of PL, PR-SRGF or FBS. **a** cPD values of BM-MSC cultured at different % of supplement from P3 to P8. PR-SRGF promotes a higher proliferation rate compared to PL and FBS resulting in a greater cPD until P8 at any supplement concentration. **b** Comparison of cPD at P5 between BM-MSC expanded in presence of PR-SRGF or PL at different % of supplement. BM-MSC expanded in presence of PR-SRGF showed a higher cPD than those expanded in PL. Statistical analysis was performed by two-way ANOVA (n = 3)

PR-SRGF promoted a higher proliferation rate compared to PL and FBS resulting in a greater cPD until P8 at any supplement concentration. In particular, at P5 the cPD of BM-MSC expanded in presence of PR-SRGF was significantly higher compared to PL at each concentration (P < 0.05 for the 5% and P < 0.01 for the 7.5 and 10% conditions). Complete data are reported in Fig. 1.

### Quantification of platelet factors released

PL and PR-SRGF were produced from two different pools of 15 donors each. The final volume of PL and PR-SRGF obtained was comparable. The concentration of PDGF-AB, PDGF-AA, PDGF-BB in PR-SRGF resulted to be respectively 5.7×, 1.7× and 2.3× higher when compared with PL. The concentrations of VEGF, EGF and TGF-β1 were comparable. Interestingly, IGF-1 was not detected in PL (Table 1).

**Table 1:** Concentration of growth factors in PL, PR-SRGF and related fold increase

Growth factor	PL	PR-SRGF	PR-SRGF/PL fold increase
VEGF, pg/ml	0.56 ± 0.15 × 10^3^	0.52 ± 0.03 × 10^3^	0.9×
EGF, pg/ml	1.65 ± 0.49 × 10^3^	1.95 ± 0.16 × 10^3^	1.2×
PDGF-AB, pg/ml	25.16 ± 6.36 × 10^3^	142.66 ± 25.25 × 10^3^	5.7×
PDGF-AA, pg/ml	4.78 ± 0.35 × 10^3^	8.30 ± 2.23 × 10^3^	1.7×
PDGF-BB, pg/ml	5.06 ± 1.24 × 10^3^	11.53 ± 1.58 × 10^3^	2.3×
TGF-β1, pg/ml	53.04 ± 12.07 × 10^3^	39.80 ± 10.10 × 10^3^	0.8×
IGF-1, ng/ml	0.00	0.11 ± 0.006 × 10^3^	–
FGF-basic, pg/ml	0.085 ± 0.008 × 10^3^	0.011 ± 0.004 × 10^3^	0.1×

Data are expressed as mean ± SD

### Immunophenotype

Expanded BM-MSC were strongly positive (≥95%) for CD90, CD44, CD105 and CD73 at P4 and P7 in presence of FBS, PL or PR-SRGF. CD31 and hematopoietic cell markers CD34 and CD45 were not expressed evidencing the isolation of homogeneous cell population.

### Immunomodulation

**Fig. 2:**
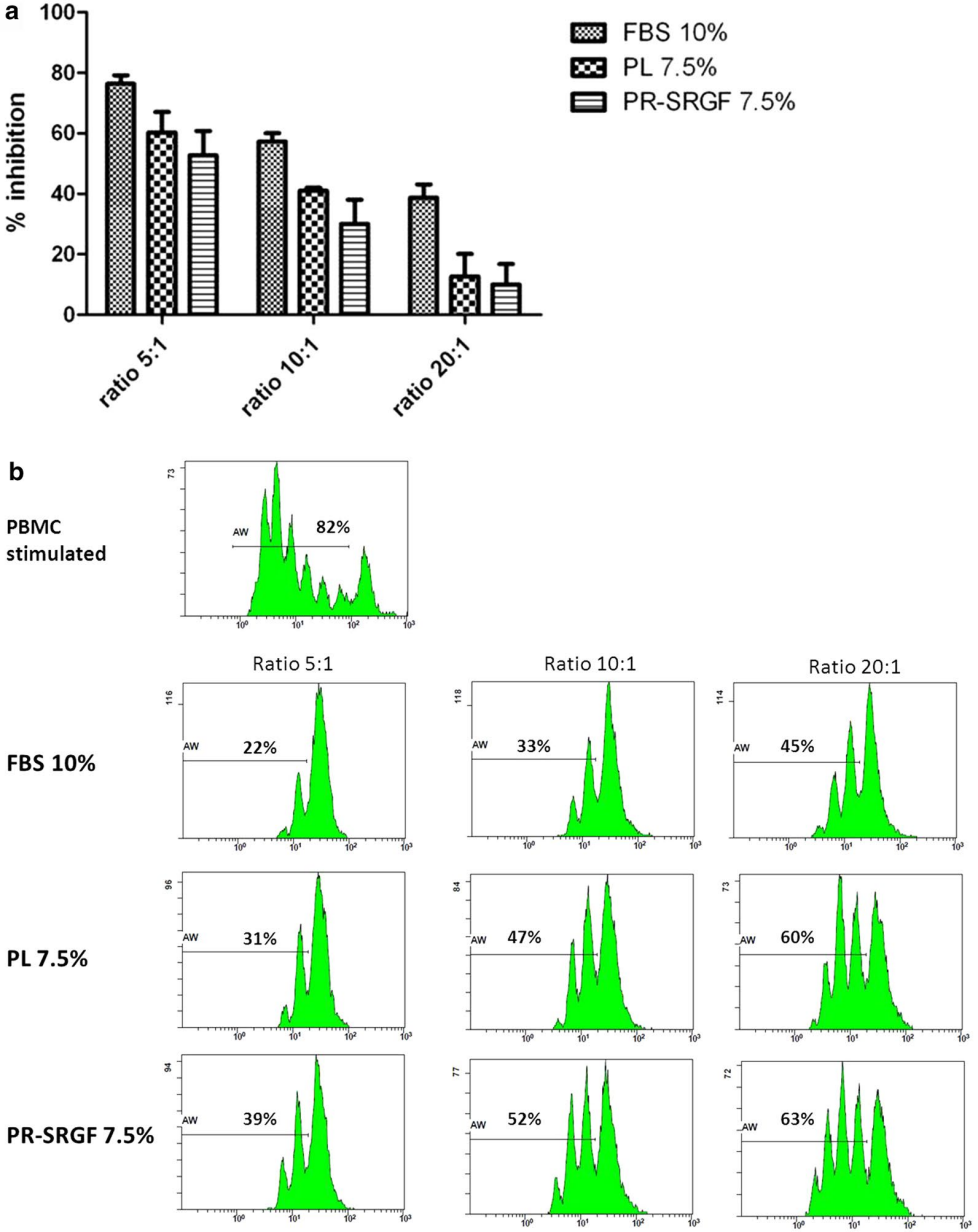
Immunomodulation properties of BM-MSC expanded in different supplements. **a** Inhibitory effect BM-MSC on CFSE labeled PBMC. Cells were co-cultured at three different PBMC:BM-MSC ratios upon PBMC stimulation. *Each bar* represents mean ± SD of % inhibition of three independent experiments. Immunomodulation of BM-MSCs expanded in PL and PR-SRGF was comparable but significantly lower when compared with FBS. Three BM-MSC batches were analyzed by two-way ANOVA. **b** PBMC proliferation was assessed by CFSE dilution method on CD45+ cells. One representative case is shown

BM-MSC expanded in 10% FBS, PL or PR-SRGF maintained their ability to inhibit proliferation of activated lymphocytes. The percentage of inhibition was directly proportional with the number of BM-MSC seeded. Immunomodulation of BM-MSCs expanded in PL and PR-SRGF was comparable but significantly lower when compared with FBS. In detail, BM-MSC expanded in FBS showed a significant difference for the ratio 20:1 versus BM-MSC expanded in PL (P < 0.01) and a significant difference for all the ratios versus BM-MSC expanded in PR-SRGF (P < 0.05) (Fig. 2).

### Tri-lineage differentiation

**Fig. 3:**
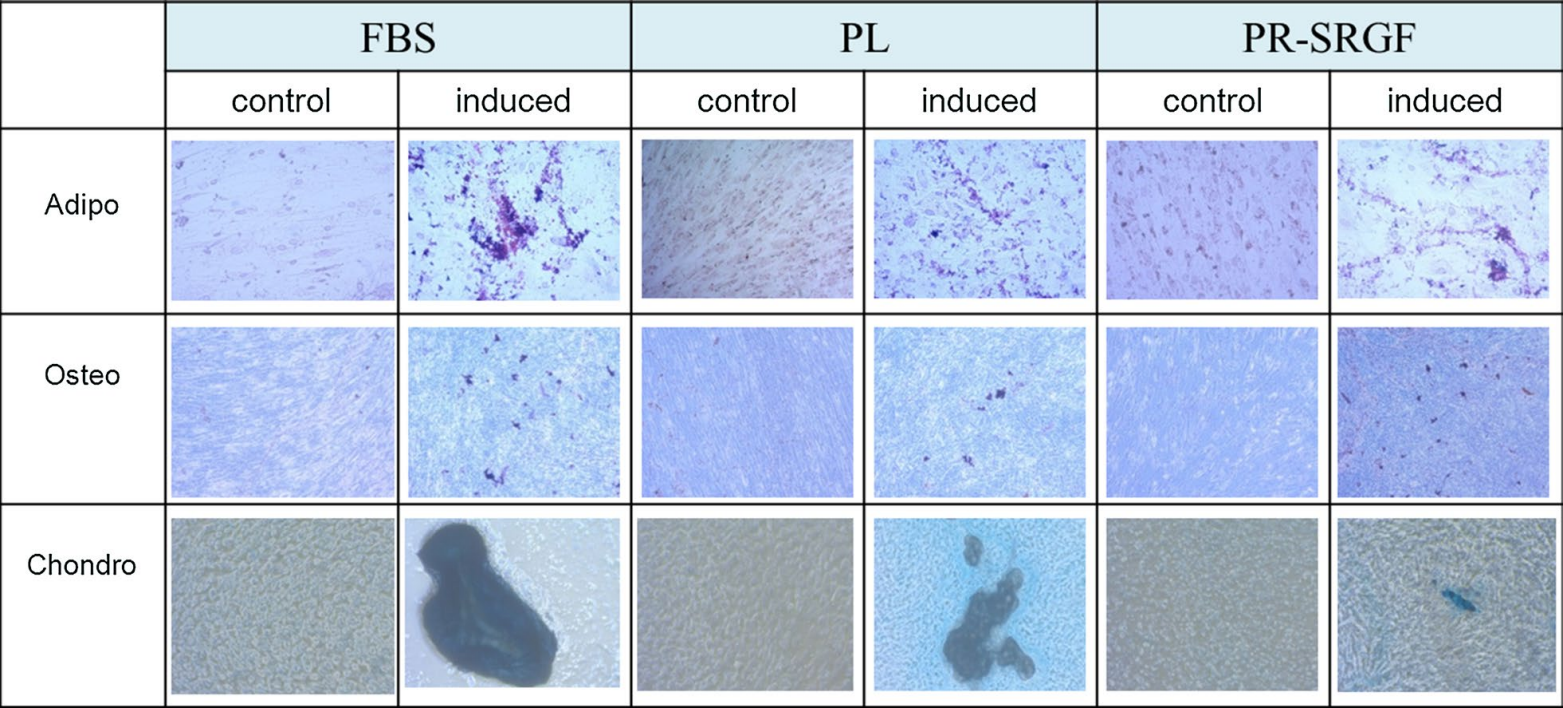
Tri-lineage differentiation. Differentiation potential into adipogenic, osteogenic and chondrogenic lineages was performed on BM-MSC cultured in presence of FBS, PL and PR-SRGF. One representative assay is reported

BM-MSC expanded at P5 in presence of 10% FBS, 7.5% PL and 7.5% PR-SRGF were able to maintain their tri-lineage differentiation potential (Fig. 3).

### Colony forming efficiency

**Fig. 4:**
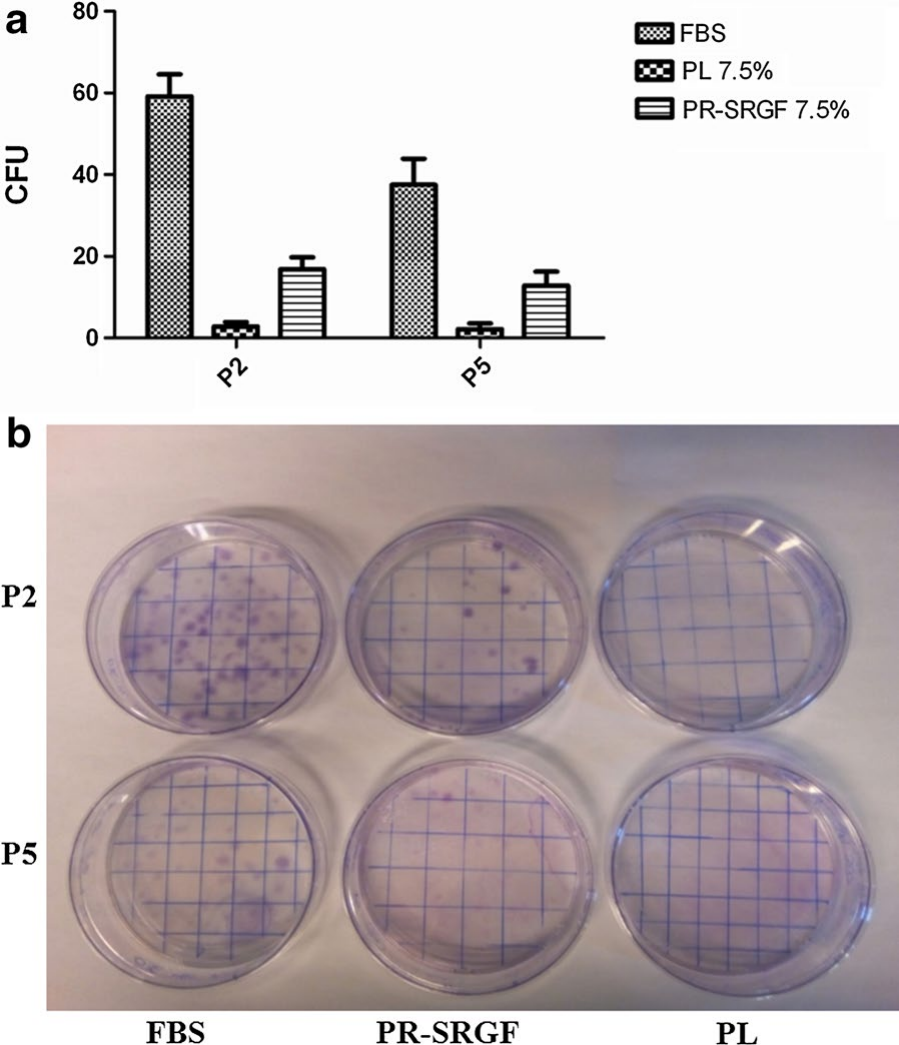
Colony forming efficiency of BM-MSC expanded in presence of the three supplements. **a** *Each bar* represents mean ± SD of CFU of three independent experiments. BM-MSC expanded in presence of FBS showed a significantly higher capacity to produce colonies than BM-MSC expanded in presence of PL and PR-SRGF at P2 and P5 (P < 0.001). Three BM-MSC batches were analyzed by two-way ANOVA test. **b** Colonies formed after plating 200 MSC in 100-mm culture dishes are shown from one representative case at P2 and P5

The colony forming efficiency of BM-MSC expanded in presence of PR-SRGF was significant higher compared to BM-MSC expanded in PL at P2 (P < 0.01) and P5 (P < 0.05). To note that BM-MSC expanded in presence of FBS showed a significantly higher capacity to produce colonies than the BM-MSC expanded in presence of PL and PR-SRGF at P2 and P5 (P < 0.001) (Fig. 4).

## Discussion

The freezing/thawing procedure is widely used to release growth factors from platelets whereas methods based on physiological activation requiring the addition of specific compounds to the platelet concentrate are not commonly applied. Nevertheless, the impact that the method used to release platelet factors could have on the composition of the final product and on the cell expansion performance has not fully investigated. It has been observed that PR obtained after platelet activation with thrombin significantly accelerated BM-MSC proliferation compared to PL obtained by freezing/thawing even if cell surface marker expression, adipogenic and osteogenic differentiation, and immunosuppressive action were similar in MSC from all culture conditions [5].

Previous works also reported that repeated freezing/thawing cycles could have negative effects on the integrity of platelet growth factors [17, 18]. Moreover, the addition of thrombin or CaCl_2_ depletes coagulation factors like fibrinogen or von Willebrand factor and adhesive proteins like fibronectin possibly influencing the composition of the final product. All these variables could result in functional differences between PL and PR.

To clarify if the method used to release growth factors could influence the cell phenotype, the ability to support the ex vivo expansion and the immunomodulatory properties of BM-MSC we have expanded the BM-MSC in presence of PL and PR-SRGF starting from a pool of 15 samples in order to reduce the between-sample variability.

We have compared the two supplements for their content in selected growth factors. It was demonstrated that PDGF and TGF-β families of cytokines are able to affect cell morphogenesis, proliferation and differentiation once released from platelets [19]. In particular, PDGF-AB/BB, TGF-β1 and FGF-basic are essential stimuli for the proliferation of MSC even if on their own these three factors are insufficient to promote MSC expansion. Therefore, other constituents of PL/PR should be present in the final product acting in synergy. In our experience, the content in growth factors resulted to be different in PL or PR-SRGF. Taking as arbitrarily relevant a fold increase ≥1.5× the concentration of PDGF-AB, PDGF-AA, PDGF-BB in PR-SRGF resulted to be higher (5.7×, 1.7× and 2.3×, respectively) compared to the concentrations found in PL. Nevertheless to understand if the observed differences could be biologically related to the expansion rate changes, further investigations are needed.

Focusing the analysis of cPD at passage 5 (this is the accepted limit for harvesting MSC cells for clinical use) we have observed that BM-MSC expanded more rapidly in 5, 7.5, 10% PR-SRGF than in PL (Fig. 1).

The immunophenotype of the cells did not show any difference of expression on the cells expanded in the three different media suggesting that at least for the surface markers that we have analyzed the phenotype was not influenced by the addition of PL or PR-SRGF and fitted with ISCT criteria regardless of the analyzed supplements at each concentration.

MSC exert potent immunosuppressive and anti-inflammatory activities [20] suppressing T cell proliferation in vitro by the production of soluble factors, including indoleamine 2,3-dioxygenase [21]. We evidenced that BM-MSC expanded in 7.5% PL or 7.5% PR-SRGF maintained a comparable ability to inhibit the lymphocyte proliferation and the percentage of inhibition was directly proportional with the number of BM-MSC seeded. It should be noted that BM-MSC expanded in 10% FBS maintained better immunomodulatory properties. This is still a matter of debate. In-fact, some authors have observed that MSC expanded in presence of PL retained their immunosuppressive capabilities [5, 22] whereas others suggested that PL-expanded MSC showed reduced immunosuppressive potential [23, 24]. Discrepancies about immunomodulation results between different research groups could reflect the different PL manufacturing process and further studies are required to clarify the matter.

We did not have observed a direct correlation between cPD and the colony forming efficiency of BM-MSC expanded in presence of FBS or platelet derivatives. In-fact, at P2 and P5 the CFU of BM-MSC expanded in presence of FBS was significantly better when compared with BM-MSC expanded in presence of PL and PR-SRGF.

Our hypothesis is that FBS, maybe due to its different composition in growth factors is able to better support the colony formation due to an improved capacity to allow the attachment of the MSC clones to the plastic surface of the petri dish whereas platelet derivatives have a higher proliferative capacity leading to an increased cell proliferation able to produce a large amount of cells in the following passages as confirmed by cPD values.

## Conclusions

We demonstrated that the method used to release platelet factors influence the composition of the product in terms of growth factor concentration and product performance. PR-SRGF obtained by platelet activation with CaCl_2_ promotes BM-MSC proliferation significantly better than PL not altering the cell phenotype and maintaining tri-lineage differentiation capacity of the expanded cells. Also the clonogenic capacity of BM-MSC expanded in PR-SRGF is better than PL. It is mandatory to understand the mechanisms that mediate the beneficial effects of platelet factors on cell growth with the aim to standardize the production process and to define adequate release criteria. This requires an effort between all the stakeholders involved in the manufacturing and use of this product.

## Abbreviations

PL: platelet lysate
PR-SRGF: platelet releasate surnatant rich in growth factors
BM-MSC: bone-marrow derived mesenchymal stromal cells
FBS: fetal bovine serum
PDGF: platelet-derived growth factor
EGF: epidermal growth factor
VEGF: vascular endothelial growth factor
FGF-basic: basic fibroblast growth factor
IGF-1: insulin like growth factor-1
TGF-β: transforming growth factor-β1
PD: population doubling
cPD: cumulative population doubling
CFSE: carboxyfluorescein succinimidyl ester
PBMC: peripheral blood mononucleated cells
7-AAD: 7-aminoactinomycin D
